# A case of recurrent epiphrenic esophageal diverticulum treated with lower esophagogastric resection and interstitial jejunal reconstruction

**DOI:** 10.1093/jscr/rjad184

**Published:** 2023-04-12

**Authors:** Ayaka Ito, Masaki Kitazono, Makoto Fujita, Naotaka Ikeda, Mayumi Eguchi, Tomohiro Oyama, Shuichiro Uchiyama, Ryoichi Toyosaki, Toyokuni Suenaga

**Affiliations:** Department of Surgery, Fujita Health University Hospital, Toyoake City, Japan; Department of Surgery, Nanpuh Hospital, Kagoshima City, Japan; Division of Medical Support, Nanpuh Hospital, Kagoshima City, Japan; Department of Surgery, Nanpuh Hospital, Kagoshima City, Japan; Department of Surgery, Nanpuh Hospital, Kagoshima City, Japan; Department of Surgery, Nanpuh Hospital, Kagoshima City, Japan; Department of Surgery, Nanpuh Hospital, Kagoshima City, Japan; Department of Surgery, Nanpuh Hospital, Kagoshima City, Japan; Department of Surgery, Nanpuh Hospital, Kagoshima City, Japan

**Keywords:** Esophageal diverticulum, Esophagogastric resection, Interstitial jejunal reconstruction

## Abstract

The patient is a 60-year-old female with a history of multiple times of recurrences of an esophageal diverticulum. She was referred for a diagnosis of persistent dysphagia and vomiting. Balloon dilation did not improve the symptoms; thus, she was referred for surgery. Esophageal fluoroscopy revealed a 5 cm diverticulum. There was no significant change in the size before and after dilation. Gastrointestinal endoscopy revealed a diverticulum in the lower esophagus, with a residue accumulation. The esophagus directly below the diverticulum was narrowed. The patient was diagnosed with recurrent lower esophageal diverticulum and underwent surgery. The operative findings showed poor coloration of the gastric fundus surrounding operated before by Nissen’s method, so the patient underwent lower esophagogastric resection and interstitial jejunal reconstruction. The postoperative course was uneventful and discharged on the 19th day. She is 6 years postoperatively and gained six kg compared to her preoperative weight. She has remained in excellent health.

## INTRODUCTION

The diverticulum of the lower esophagus and epiphrenic diverticula is rare. Epiphrenic esophageal diverticula are often the result of an esophageal motor abnormality, such as a lack of coordination between the distal esophagus and lower esophageal sphincter (LES) [1, 2]. As the diverticulum enlarges, the passage of food becomes difficult and may cause aspiration, necessitating surgery. We report a case in which lower esophageal diverticulum recurrence was successfully treated with lower esophagogastric resection and interstitial jejunal reconstruction.

## CASE REPORT

**Figure 1:**
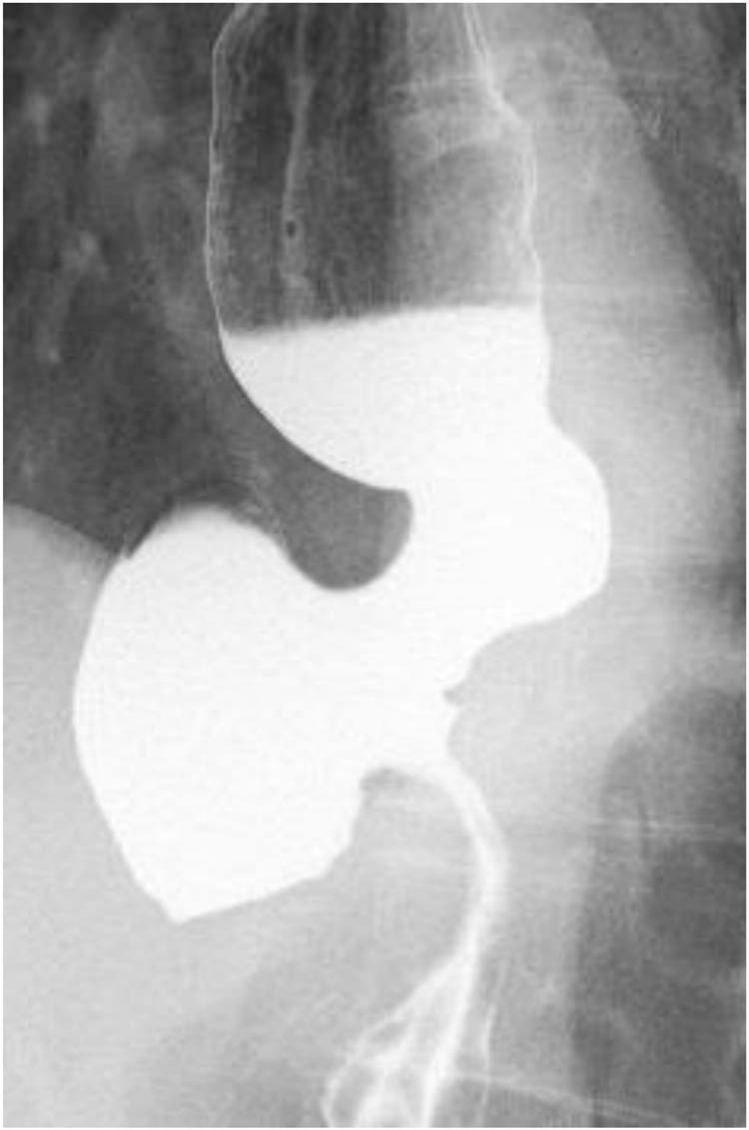
Esophageal fluoroscopy (before dilation). There was a 5 cm-sized diverticulum in the lower esophagus just above the eruption.

**Figure 2:**
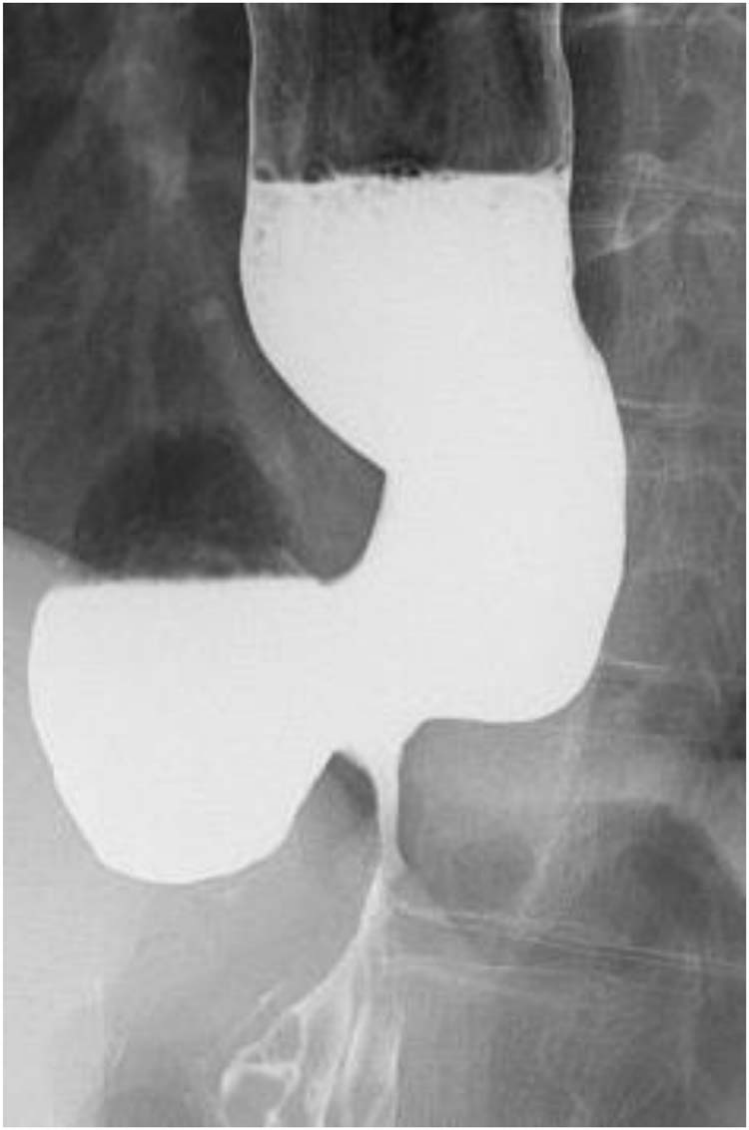
Preoperative esophageal fluoroscopy (after dilation). There was no significant change compared to the pre-expansion period.

**Figure 3:**
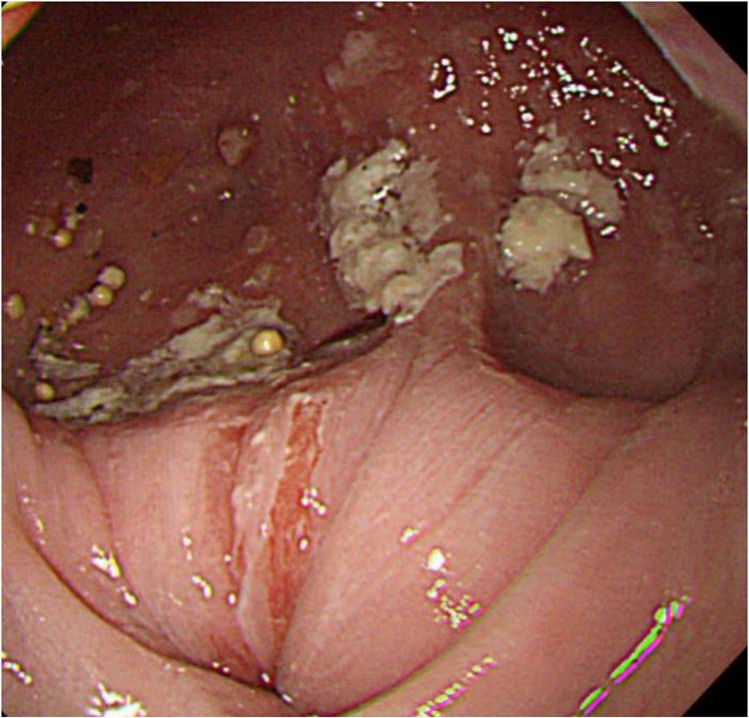
Upper digestive tube endoscopy. The esophagus just below the diverticulum was stenotic, and there was residue accumulation in the diverticulum.

A 60-year-old female with dysphagia and vomiting visited the university hospital. In 2007, she underwent esophageal diverticulum resection (procedure unknown) with a diagnosis of an esophageal diverticulum, which subsequently recurred. In 2011, she underwent a transesophageal hiatus diverticulectomy and a fundoplication (Nissen procedure) at a local hospital. In 2013, dysphagia and vomiting appeared. The patient was diagnosed with recurrence and underwent balloon dilatation for follow-up. In 2015, the patient came to our hospital due to persistent symptoms. After one balloon dilation, the patient did not show any improvement and was referred for surgery. Esophageal fluoroscopy revealed a diverticulum 5 cm in size in the lower esophagus just above the eruption. There was no significant change in the diverticulum size before and after dilation (Figs 1 and 2). Gastrointestinal endoscopy revealed a diverticulum in the lower esophagus, with a residue accumulation (Fig. 3). The esophagus directly below the diverticulum was narrowed. The patient was diagnosed with recurrent lower esophageal diverticulum and underwent surgery.

**Figure 4:**
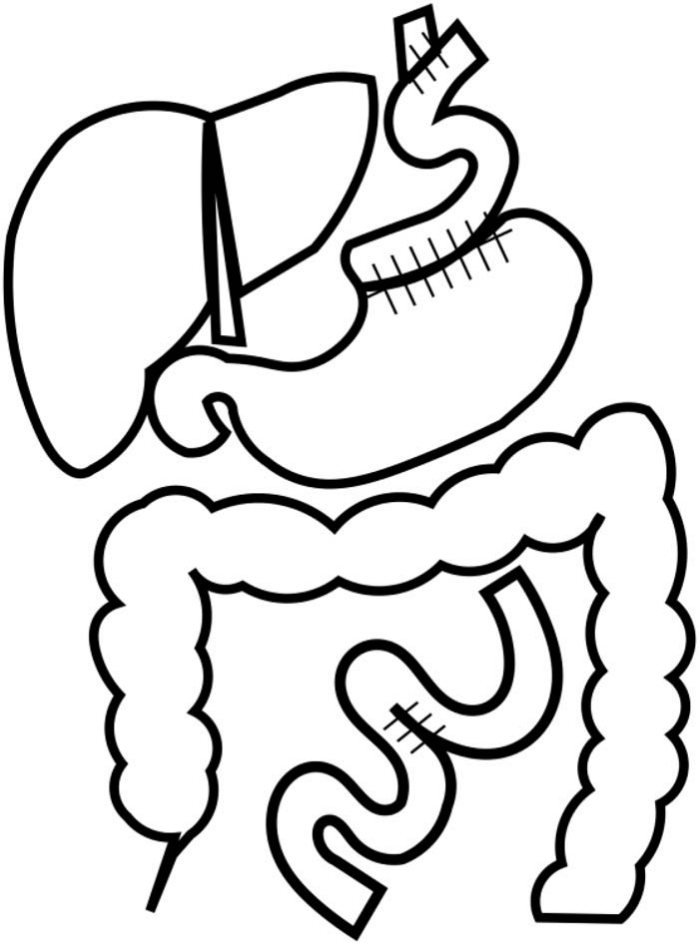
Reconstruction. The patient underwent lower esophagogastric resection and interstitial jejunal reconstruction due to poor coloration of the vault capsule by Nissen’s method.

**Figure 5:**
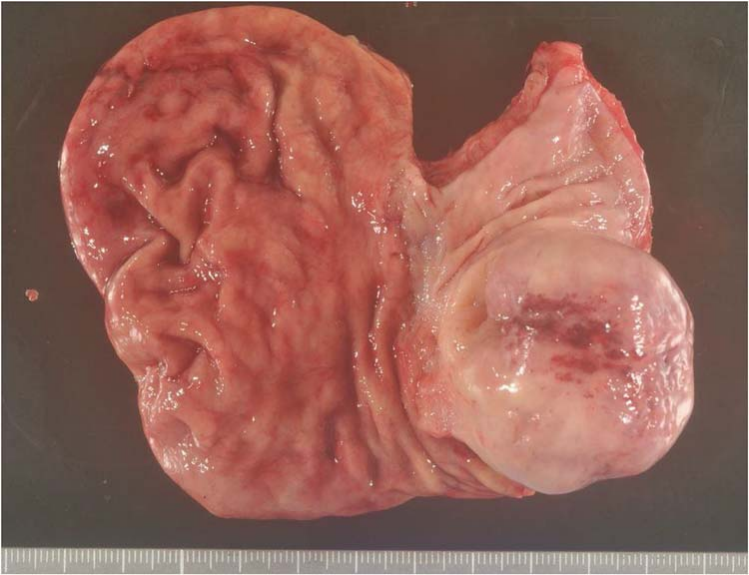
Excised specimen. Diverticulum-like cyst formation was observed in the esophago-gastric junction due to esophageal mucosal dilatation and entrapment of the esophageal mucosa associated with outer membrane adhesion.

**Figure 6:**
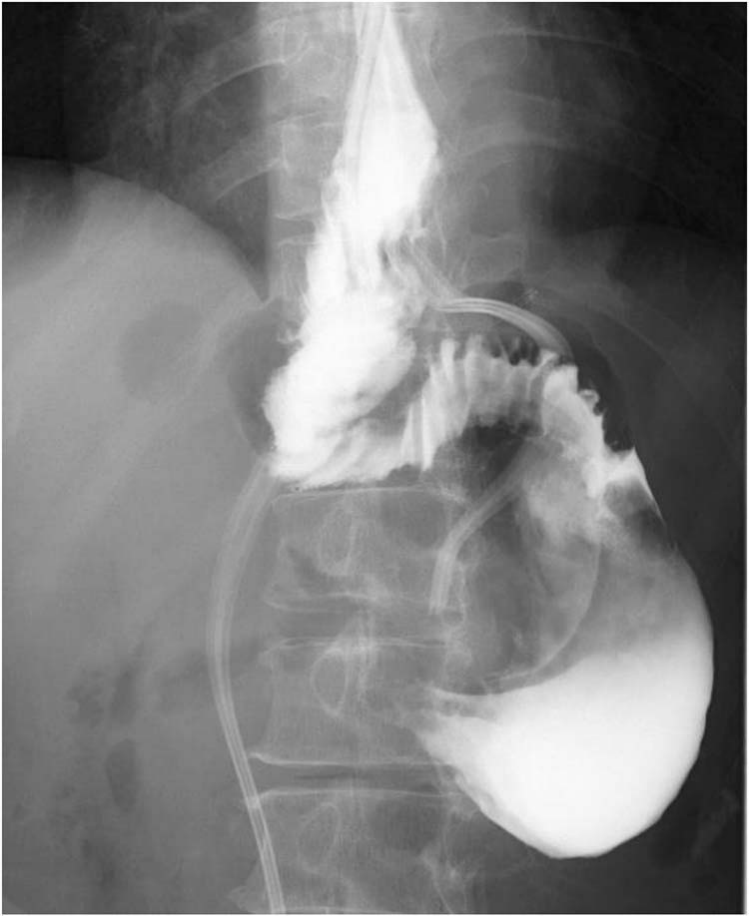
Postoperative esophageal fluoroscopy. No stenosis findings or stagnation of contrast media was observed.

The operative findings showed poor coloration of the vault surrounding by Nissen’s method, so the patient underwent lower esophagogastric resection and interstitial jejunal reconstruction (Fig. 4). The operation time was 230 minutes, and the blood loss was 580 ml. The resection specimen showed diverticulum-like cystic formation due to the esophageal mucosal dilatation at the esophagogastric junction and the entrapment of the esophageal mucosa associated with the outer membrane adhesion (Fig. 5). The postoperative course was generally favorable. Esophageal fluoroscopy on the seventh postoperative day showed no evidence of stricture. The contrast medium drained into the stomach without stagnation (Fig. 6). On the 13th postoperative day, she developed dumping-like symptoms, which improved with dietary guidance. Besides that, the patient had no other significant problems and was discharged from the hospital on the 19th day. She is now six years postoperatively and has gained 6 kg compared to her preoperative weight. The patient is doing well.

## DISCUSSION

The cause of the lower esophageal diverticulum is believed to be related to increased intraesophageal pressure, which is often associated with esophageal achalasia and esophageal spasm. The diverticulum is usually located in the distal 10 cm of the esophagus, with herniation of mucosa and submucosa through the muscularis propria, toward the right in the mediastinum [3].

Most patients with epiphrenic diverticula are asymptomatic, the remainder presenting with regurgitation, dysphagia, nocturnal cough or pulmonary symptoms and weight loss [4]. It is important to appreciate that symptoms are often a reflection of the underlying esophageal motor disorder rather than the diverticulum itself. Indeed, it is well recognized that diverticular size bears little relation to symptomatology [5].

The esophageal diverticulum is rarely operated on because many cases are asymptomatic. There are a wide variety of surgical procedures for esophageal diverticulum. The basic technique is resection of the diverticulum, with the addition of myomectomy or diverticuloplasty [6]. At this point, there is still much debate as to which technique should be chosen [7].

On the other hand, if we opt for surgical resection, we must choose between a transabdominal and a transthoracic approach. More recently, it has become common to combine endoscopic techniques as minimal invasive procedures. Advantages of the laparoscopic transhiatal approach include two advantages: first, optimal visualization of both the EGJ and lower mediastinum to perform a long myotomy; and second, perfect alignment of the stapler to the longitudinal axis of the esophagus and the possibility of construction of a fundoplication [8]. Soares *et al*. reported a significantly shorter median length of stay in the laparoscopic group compared to the thoracotomy group [9]. The laparoscopic transhiatal approach for achalasia has also been shown to result in lower conversion rates, shorter hospital stays, improved dysphagia and a reduced reverse flow compared to the transthoracic approach [10, 11].

In our case, we could not choose an endoscopic approach because it was the third reoperation, but chose a transhiatal approach with an open abdomen. Valentini *et al*. also reported a case of early recurrence after myotomy for the esophageal diverticulum [12]. Therefore, they attempted another diverticulectomy again. In the present case, the esophageal diverticulum was resected twice, but the diverticulum recurred. In such a case, lower esophagogastric resection and interstitial jejunal reconstruction were performed, and the patient had a good prognosis, which we considered an effective reconstruction method.

## CONCLUSION

This case demonstrated the following two points. First, lower esophagogastric resection and interstitial jejunal reconstruction are effective methods of reconstruction for the lower esophageal diverticulum. In addition, it is necessary to remember that diverticular resection may result in recurrence when selecting a technique.

## Data Availability

Data sharing is not applicable to this article as no new data were created or analyzed in this study.
